# Whole exome sequencing in systemic juvenile idiopathic arthritis

**DOI:** 10.1186/1546-0096-13-S1-O2

**Published:** 2015-09-28

**Authors:** F Moghaddas, D De Nardo, P Baker, L Gordon, S Sadedin, A Oshlack, J Akikusa, R Allen, J Munro, J Ellis, S Masters

**Affiliations:** 1 The Walter and Eliza Hall Institute of Medical Research, Inflammation, Parkville, Australia; 2 The University of Melbourne, Medical Biology, Melbourne, Australia; 3 Murdoch Children's Research Institute, Bioinformatics, Melbourne, Australia; 4 The Royal Children's Hospital, Paediatric Rheumatology, Melbourne, Australia; 5 Murdoch Children's Research Institute, Population Health, Melbourne, Australia

## Introduction

Systemic juvenile idiopathic arthritis (sJIA) shares clinical features with classic monogenic autoinflammatory diseases, characterised by fevers, arthritis and evanescent rashes. Disease exacerbations are associated with elevated serum cytokine levels including 1L-1β, IL-6, and IL-18; and clinical response to anakinra, canakinumab and tocilizumab suggests that cytokine dysregulation is a key pathophysiological mechanism. Macrophage activation syndrome (MAS) may complicate sJIA, rendering individuals clinically indistinguishable from their familial haemophagocytic lymphohistiocytosis (fHLH) counterparts; with NK and CD8+ cell dysfunction leading to sustained immune cell activation and cytokine storm. Whilst there have been HLA associations and polymorphisms noted on sJIA genome-wide association studies, in rare cases mutations have been found in genes encoding key components of the inflammatory response, which may contribute to disease pathogenesis.

## Objectives

We aim to identify rare variants in innate immune genes that contribute to cytokine imbalance and lead to the inflammatory phenotype seen in sJIA patients.

## Methods

We performed whole exome sequencing on probands with sJIA and their parents (trios). Recessive, compound heterozygous and de novo inheritance models were tested and variants evaluated for potential functional effect using a combination of SIFT, PolyPhen and MutationTaster. We confirmed candidate pathogenic variants by Sanger sequencing and cross-checked these variants with the Infevers database, known fHLH causing mutations and inflammatory pathways. We harnessed CRISPR-Cas9 gene editing technology to model mutations in appropriate cell lines, and investigate the pathogenic nature of identified novel variants.

## Results

Exome sequencing was performed on a total of 16 trios and unaffected siblings, with data from 13 trios suitable for analysis. There were no rare variants or indels inherited in a dominant, recessive or compound heterozygous manner, and predicted to affect function in a known inflammatory pathway. Furthermore, rare variants with predicted functional effects in Infever genes or fHLH genes were not identified. In total, there were 26 de novo variants (median = 2, range 0 to 6 per trio), with 17 of these predicted to affect protein function (median = 1, range 0 to 2 per trio). At least one de novo variant affects a known innate immune gene, and the pathogenic nature of this variant is being confirmed in vitro.

## Conclusion

Disease in a small subset of patients with sJIA may be accounted for by rare de novo variants, however this requires further in vitro confirmation. Alternatively, it is possible that some of these individuals may in fact represent a separate disease entity.

